# A thorough analysis of data on the correlation between COL9A1 polymorphisms and the susceptibility to congenital talipes equinovarus: a meta-analysis

**DOI:** 10.1186/s13018-024-04834-5

**Published:** 2024-06-10

**Authors:** Mohammad Golshan-Tafti, Seyed Alireza Dastgheib, Kamran Alijanpour, Reza Bahrami, Mahta Mazaheri, Hossein Neamatzadeh

**Affiliations:** 1 Department of Pediatrics, Islamic Azad University of Yazd, Yazd, Iran; 2 Department of Medical Genetics, School of Medicine, Shiraz University of Medical Sciences, Shiraz, Iran; 3 General Practitioner, Babol University of Medical Sciences, Babol, Iran; 4 Neonatal Research Center, Shiraz University of Medical Sciences, Shiraz, Iran; 5 Mother and Newborn Health Research Center, Shahid Sadoughi University of Medical Sciences, Yazd, Iran

**Keywords:** Congenital clubfoot, Talipes equinovarus, CTEV, Foot deformities, COL9A1, Polymorphism

## Abstract

**Background:**

Congenital talipes equinovarus (CTEV) is a prevalent pediatric deformity with a multifactorial etiology. The objective of this meta-analysis was to explore the association between genetic variations in COL9A1 and the susceptibility to CTEV.

**Methods:**

A comprehensive analysis of pertinent literature released before November 15, 2023, in electronic bibliographic databases was carried out. The importance of the connection was clarified through odds ratios (ORs) with 95% confidence intervals (CIs), utilizing random or fixed-effects models depending on study heterogeneity. Statistical analysis was executed using Comprehensive Meta-Analysis software (Version 4.0).

**Results:**

A total of eight case-control studies involving 833 CTEV patients and 1280 healthy individuals were included in the analysis. Among these, four studies investigated the rs1135056 variant, encompassing 432 CTEV cases and 603 controls; two studies examined the rs35470562 variant, with 189 CTEV cases and 378 controls; and two studies explored the rs592121 variant, including 212 CTEV cases and 299 controls. The results revealed a significant association between the rs1135056 and rs35470562 polymorphisms in the COL9A1 gene, suggesting an increased risk of CTEV in the overall population. Conversely, no such association was found for the rs592121 variant.

**Conclusion:**

Our findings reveal a substantial association between the genetic variants COL9A1 rs1135056 and rs35470562 and susceptibility to CTEV. Conversely, the variant rs592121 did not exhibit any corresponding link. However, the limitations imposed by the small study population have compromised the statistical reliability and generalizability of the results.

## Introduction

Congenital talipes equinovarus (CTEV), commonly referred to as clubfoot, is a prevalent musculoskeletal anomaly in the pediatric population, with an estimated birth prevalence of approximately 1 in 1,000 individuals [1, 2]. The clinical presentation of CTEV includes adduction of the forefoot, plantar flexion of the ankle, excessive inward rotation of the tibia, and overpronation of the calcaneus [3]. While CTEV is typically idiopathic in nature, it can also be associated with other congenital malformations, such as arthrogryposis, myelomeningocele, spina bifida, tethered cord syndrome, and amniotic band syndrome [4–6]. The global incidence of CTEV is influenced by the birth rate, with an estimated 100,000 cases reported annually worldwide. Furthermore, bilateral involvement is observed in approximately 30–50% of cases [7, 8]. It is noteworthy that a significant proportion of affected individuals reside in low- and middle-income countries, particularly in Southeast Asia and Africa, where access to appropriate treatment options may be limited [8–10]. The higher incidence of CTEV in these regions is postulated to have a genetic basis [11, 12]. Over the past few decades, non-surgical interventions, specifically the Ponseti technique, have become the preferred approach to managing CTEV. This technique involves a series of manipulations and castings, followed by Achilles tenotomy performed percutaneously, and the subsequent use of a foot abduction brace to minimize the need for additional surgical procedures [13, 14]. The occurrence of CTEV has been reported to vary across different geographical regions, with the lowest prevalence documented among individuals of Chinese descent and the highest among those of Polynesian ethnicity [11, 15, 16]. Additionally, the female-to-male ratio in CTEV cases remains consistent across diverse ethnic groups, ranging from approximately 1:2.0 to 1:2.5 [1, 17]. Moreover, developmental dysplasia of the hip (DDH) is frequently observed in syndromic malformations, such as clubfoot, due to its impact on connective tissue [18]. This condition is linked to various complications, including instability, dislocation, and the early onset of osteoarthritis in the hip joint [19]. Investigating the correlation between clubfoot and DDH is an area of ongoing exploration and clinical significance. Although these congenital conditions may seem unrelated, emerging data suggests a potential connection, with certain studies indicating a higher occurrence of DDH among individuals with clubfoot [18, 19]. The precise mechanisms underlying this correlation are not yet fully understood, but researchers have proposed several theories, including shared genetic and environmental factors, as well as possible biomechanical interactions during prenatal development.

The etiology of CTEV, although speculative in nature, is widely believed to be multifactorial in origin [17, 20]. While genetics undoubtedly play a significant role, various environmental factors also exert influence on the development of CTEV [21–23]. Notably, maternal smoking, advanced maternal age, and maternal nutrition have all been linked to an increased risk of CTEV. Moreover, it is worth noting that the interplay between genetic and environmental factors can contribute to the observed phenotypic variability among individuals with CTEV, underscoring the importance of a multifactorial model in understanding the etiology of clubfoot [8, 17, 24]. Numerous studies have indeed demonstrated the substantial involvement of genetic factors in the development of CTEV. Although the exact mode of inheritance remains elusive, the recurrence patterns observed in families strongly suggest a multifactorial genetic basis characterized by both inherited and sporadic genetic mutations [25, 26]. However, it is important to acknowledge that research on the susceptibility genes of CTEV has made limited progress, and no specific gene has yet been identified as the CTEV gene [27, 28]. The heritability of CTEV is estimated to be approximately 65%, yet the precise patterns of heredity and penetrance remain unclear. Approximately 25% of all cases exhibit familial tendencies, and a sibling of an affected child has a 2–4% chance of developing CTEV [1, 29–31]. Furthermore, data from twin studies have revealed higher concordance rates among monozygotic twins (33%) compared to dizygotic twins (3%), indicating a more prominent role for heritability in the development of CTEV [13]. Presently, only one genome-wide association study (GWAS) has been conducted on CTEV, which identified genetic variants in the NCOR2 and ZNF664 genes as being associated with the pathogenesis of CTEV in non-Hispanic and European descent individuals with CTEV [32, 33].

The study of the genetics of CTEV is a captivating field of research. In humans, genetic variations in collagen IX genes are connected to the functional state of joint cartilages, while genetic abnormalities in collagen formation are associated with short stature, severe skeletal deformities, and chondrodysplasia [34]. Collagen IX is a heterodimer molecule composed of identical chains of α1, α2, and α3 (α1(IX)–α3(IX)), which are encoded by the genes collagen type 9 alpha 1 (COL9A1), alpha 2 (COL9A2), and alpha 3 (COL9A3) located on chromosome locus 6q13, respectively [35, 36]. Through the candidate cloning strategy, the COL9A1 gene in the candidate region was initially confirmed as an associated gene with the risk of CTEV. Furthermore, the transmission disequilibrium test analysis demonstrated a linkage between the COL9A1 gene and a predisposition to CTEV [37]. In abductor hallucis muscle samples of CTEV cases, an increased expression of COL9A1 was observed, indicating that the COL9A1 gene may be a significant predisposition gene for CTEV [38, 39]. Convincing evidence from case-control and family-based linkage studies has indicated an association between COL9A1 polymorphisms and the risk of CTEV. However, due to the sample sizes of individual studies, the results continue to be debatable. To the best of our knowledge, no relevant meta-analysis has examined the association of COL9A1 polymorphisms with the risk of CTEV. Therefore, the objective of this meta-analysis is to determine the association between genetic variants at the COL9A1 gene and the risk of CTEV.

## Materials and methods

### Search approaches

The ethical endorsement was not obligatory for the present investigation, as it constitutes a systematic review and meta-analysis. This study was conducted in accordance with the guidelines outlined in the Preferred Reporting Items for Systematic Reviews and Meta-Analyses. The bibliographic databases, including PubMed, Web of Science, Europe PMC, ResearchGate, Elsevier, Cochrane Library, EMBASE, SciELO, Google Scholar, Chinese National Knowledge Infrastructure (CNKI), Wanfang Data Company, Chaoxing, China/Asia On Demand (CAOD), Chinese Medical Citation Index (CMCI), VIP Information Consulting Company (VIP), Chinese Medical Current Contents (CMCC), Chinese Biomedical Database (CBD), and Weipu Periodical Database, were utilized to search for relevant studies that assessed the correlation between COL9A1 polymorphisms and the risk of Congenital Talipes Equinovarus (CTEV). The search period spanned from the inception of the databases until November 15, 2023. The search was conducted using a combination of specific keywords and Medical Subject Headings (MeSH) terms, including ‘Congenital Talipes Equinovarus’, ‘Talipes Equinovarus’, ‘CTEV’, ‘Clubfoot’, ‘Collagen alpha-1(IX)’, ‘COL9A1’, ‘Gene’, ‘Association’, ‘Correlation’, ‘Genetic’, ‘Predisposition’, ‘Polymorphism’, ‘DNA Sequence’, ‘Single-Nucleotide Polymorphism’, ‘SNPs’, ‘Genotype’, ‘Frequency’, ‘Mutation’, ‘Mutant’, ‘Allele’, ‘Variation’, and ‘Variant’. Additionally, a manual search of the references cited in the retrieved articles and reviews was conducted to identify any other relevant publications. The search was limited to human studies and did not impose any language restrictions.

### Including and excluding criteria

All studies included in the analysis met the following criteria: (1) they were original studies with either a case-control or cohort design, published in various languages; (2) they investigated the correlation between COL9A1 polymorphisms and the risk of CTEV; (3) they provided sufficient and accessible data to calculate the odds ratios (ORs) and 95% confidence intervals (CIs). The primary exclusion criteria were: (1) studies without a control group; (2) case reports, case series, commentaries, letters, editorials, reviews, animal experiments, in vitro cell experiments, oral presentations, and previous meta-analyses; (3) studies with incomplete literature data or where the original text could not be obtained after contacting the original author; (4) inadequate or inaccessible data for analysis; (5) overlapping or duplicate data from the same population.

### Data extraction

Two investigators independently examined the bibliographies, gathered data, and cross-referenced them according to the aforementioned inclusion and exclusion criteria. In case of discrepancies, resolution would be sought through debate or a meeting involving a third researcher. Additionally, contact with the original author via email would be made when deemed necessary. During the literature screening process, the title and abstract were initially reviewed to eliminate obviously irrelevant sources. Subsequently, the full text was carefully examined to determine eligibility for inclusion. The literature that met the required standards yielded the following key information: the first author’s name, ethnicity, publication date, country of origin, genotyping methods, the total number of cases and controls for CTEV, genotype frequencies of CTEV cases and healthy controls for each available COL9A1 polymorphism, Hardy-Weinberg equilibrium (HWE) test results, and minor allele frequencies (MAFs) in the healthy control group. In cases where multiple studies were conducted by the same investigator(s) and involved duplicated or overlapping records, only the most recent published data or the study with the largest sample size was considered for incorporation into the analysis.

### Statistical analysis

The HWE in healthy subjects for each individual study was assessed using the Pearson’s chi-square Statistic, employing free online software. A *p*-value below 0.05 was deemed to be statistically significant. The association between COL9A1 polymorphisms and susceptibility to CTEV was quantified using odds ratios (ORs) with 95% confidence intervals (CIs). To determine the statistical significance of the combined data, the Z-test was applied to ascertain the difference between the population mean and sample mean [40]. This meta-analysis incorporated five genetic models, including allelic (B vs. A), heterozygote (BA vs. AA), homozygote (BB vs. AA), recessive (BB vs. BA + AA), and dominant (BB + BA vs. AA). The chi-square test is commonly utilized to evaluate the significance of heterogeneity, with a significance level of *p* < 0.05. Furthermore, based on Cochrane, the level of heterogeneity between studies was classified on a scale of 0 to 100% [41, 42]. The degree of heterogeneity was quantified using the I^2^ index, which represents the proportion of total variation between studies attributable to their distinct characteristics. Therefore, if I2 exceeded 50%, a random-effect model (DerSimonian-Laird method) was employed. Otherwise, fixed-effect models (Mantel-Haenszel method) were utilized for interpretation [42, 43]. Sensitivity analysis was performed by systematically excluding one study at a time to assess the robustness of the findings. To assess publication bias, Begg’s test was employed by plotting the standard error of each study against its odds ratio, and Egger’s test was conducted alongside visual examination of the funnel plot for asymmetry. If publication bias was detected, the trim-and-fill method was employed to adjust the conclusions accordingly [44, 45]. The primary studies were synthesized using the Comprehensive meta-analysis software (Version 4.0) developed by Biostat, USA. A *p*-value below 0.05 on either end was deemed to possess statistical importance.

## Results

### Study characteristics

**Fig. 1:**
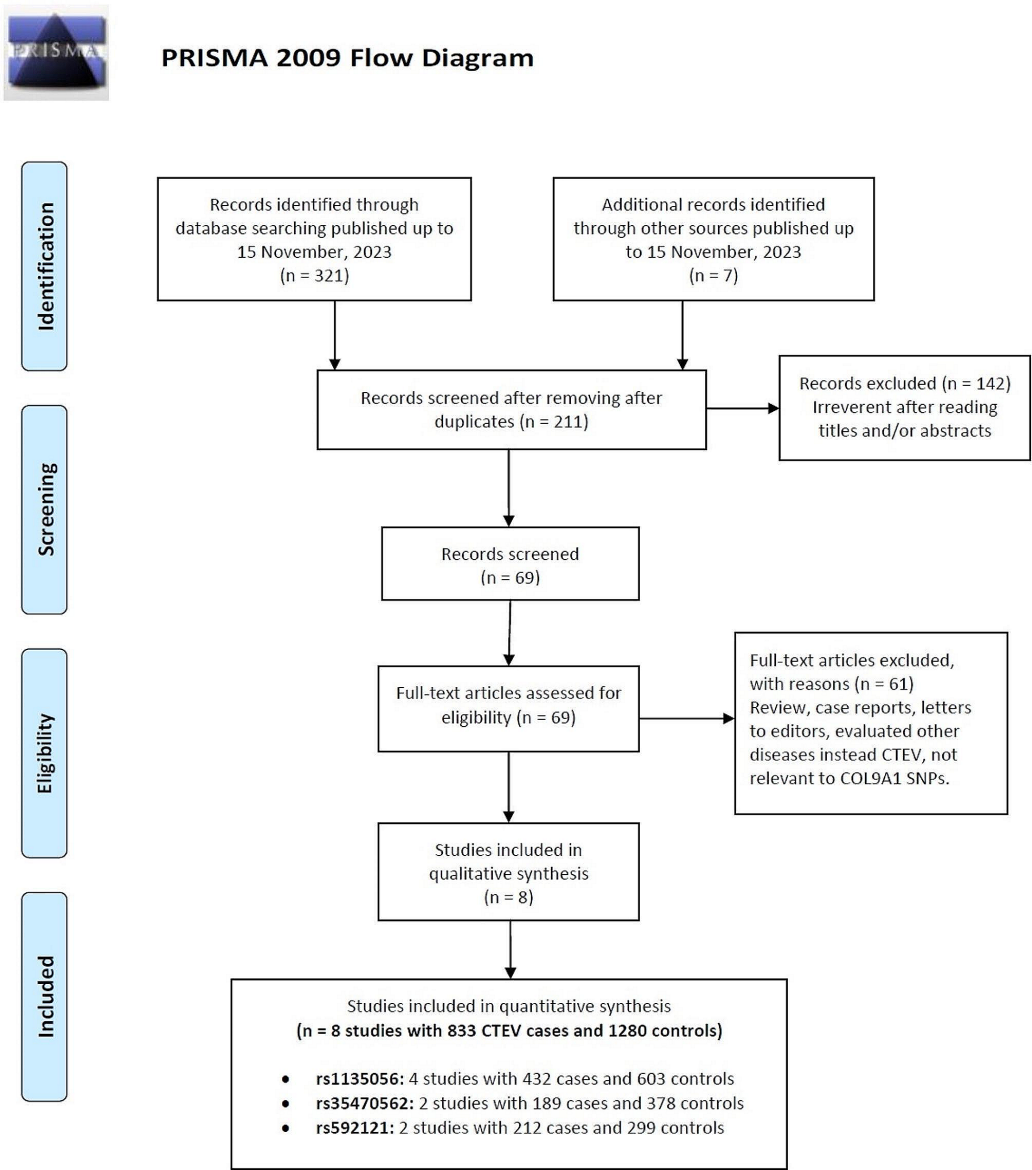
Flow diagram of the study selection process

Detailed flowcharts illustrating the complex selection process are presented in Fig. 1. Initially, a comprehensive search was conducted across multiple electronic databases, accompanied by a thorough manual review of the complete contents on a page-by-page basis, leading to the identification of 328 papers. Subsequently, by carefully evaluating the research titles and abstracts, 117 duplicate documents and 142 irrelevant articles were eliminated, resulting in 69 articles for detailed examination. Ultimately, a total of eight case-control studies, involving 833 cases with CTEV and 1280 healthy individuals, were selected, meeting our inclusion criteria, as detailed in four publications on the rs1135056 (c.1862 A > G, p.Gln621Arg), rs35470562 (c.2050G > A, p.Glu441Lys), and rs592121 (c.1015T > C, p.Ser339Pro) genetic variations. Among these investigations, four focused on rs1135056, with 432 CTEV cases and 603 controls, while two studies examined rs35470562, including 189 CTEV cases and 378 controls, and another two studies investigated rs592121, encompassing 212 CTEV cases and 299 controls. The selected studies were published between 2011 and 2022 in English and Chinese, and were conducted on populations of Asian descent in China and India. The genotyping technique employed for assessing the genetic variations in all studies included in this analysis utilized the polymerase chain reaction restriction fragment length polymorphism (PCR-RFLP) method. It is noteworthy that the genotype distribution for rs1135056 in the control cohort within a study deviated from Hardy-Weinberg Equilibrium principles (*p* = 0.005). Furthermore, Table 1 delineates the frequencies of the minor allele for each genetic variation examined in this investigation within the control cohort.

**Table 1:** Characteristics of the studies included in meta-analysis

First Author/Year	Ethnicity (Country)	Genotyping Methods	SOC	Case/Control Number	Patients					Control					MAFs	HWE
					Genotypes			Alleles		Genotypes			Alleles			
**rs1135056**					**AA**	**AG**	**GG**	**A**	**G**	**AA**	**AG**	**GG**	**A**	**G**		
Liu 2011	China(Asian)	PCR-RFLP	HB	118/100	34	58	26	126	110	48	33	19	129	71	0.335	0.005
Zhao 2016	China(Asian)	PCR-RFLP	HB	87/174	32	37	18	101	73	74	73	27	221	127	0.365	0.210
Li 2016	China(Asian)	PCR-RFLP	HB	102/204	34	50	18	118	86	90	93	21	273	135	0.331	0.673
Raikwar 2022	India(Asian)	PCR-RFLP	NA	125/125	78	32	15	188	62	55	54	16	164	86	0.344	0.632
**rs35470562**					**GG**	**GA**	**AA**	**G**	**A**	**GG**	**GA**	**AA**	**G**	**A**		
Zhao 2016	China(Asian)	PCR-RFLP	HB	87/174	30	41	16	101	73	78	80	16	236	112	0.322	0.482
Li 2016	China(Asian)	PCR-RFLP	HB	102/204	98	4	0	200	4	197	7	0	401	7	0.017	0.803
**rs592121**					**TT**	**TC**	**CC**	**T**	**C**	**TT**	**TC**	**CC**	**T**	**C**		
Zhao 2016	China(Asian)	PCR-RFLP	HB	87/174	28	40	19	96	78	60	80	34	200	148	0.425	0.432
Raikwar 2022	India(Asian)	PCR-RFLP	NA	125/125	54	57	14	165	85	55	59	11	169	81	0.324	0.386

Abbreviations: PCR-RFLP: polymerase chain reaction restriction fragment length polymorphism; SOC: Source of Controls, HB: Hospital Based; PB: Population Based; HWE: Hardy-Weinberg equilibrium; MAF: Minor Allele Frequency

### Quantitative synthesis

**Fig. 2:**
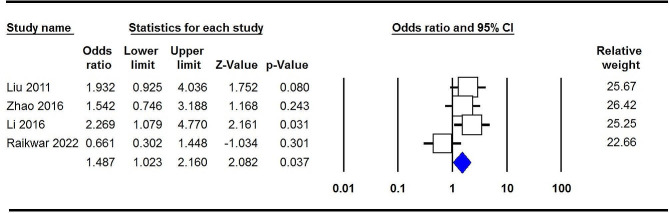
Forest plots for the association of COL9A1 rs1135056 polymorphism and risk of CTEV under the homozygote model (GG vs. AA)

**Fig. 3:**
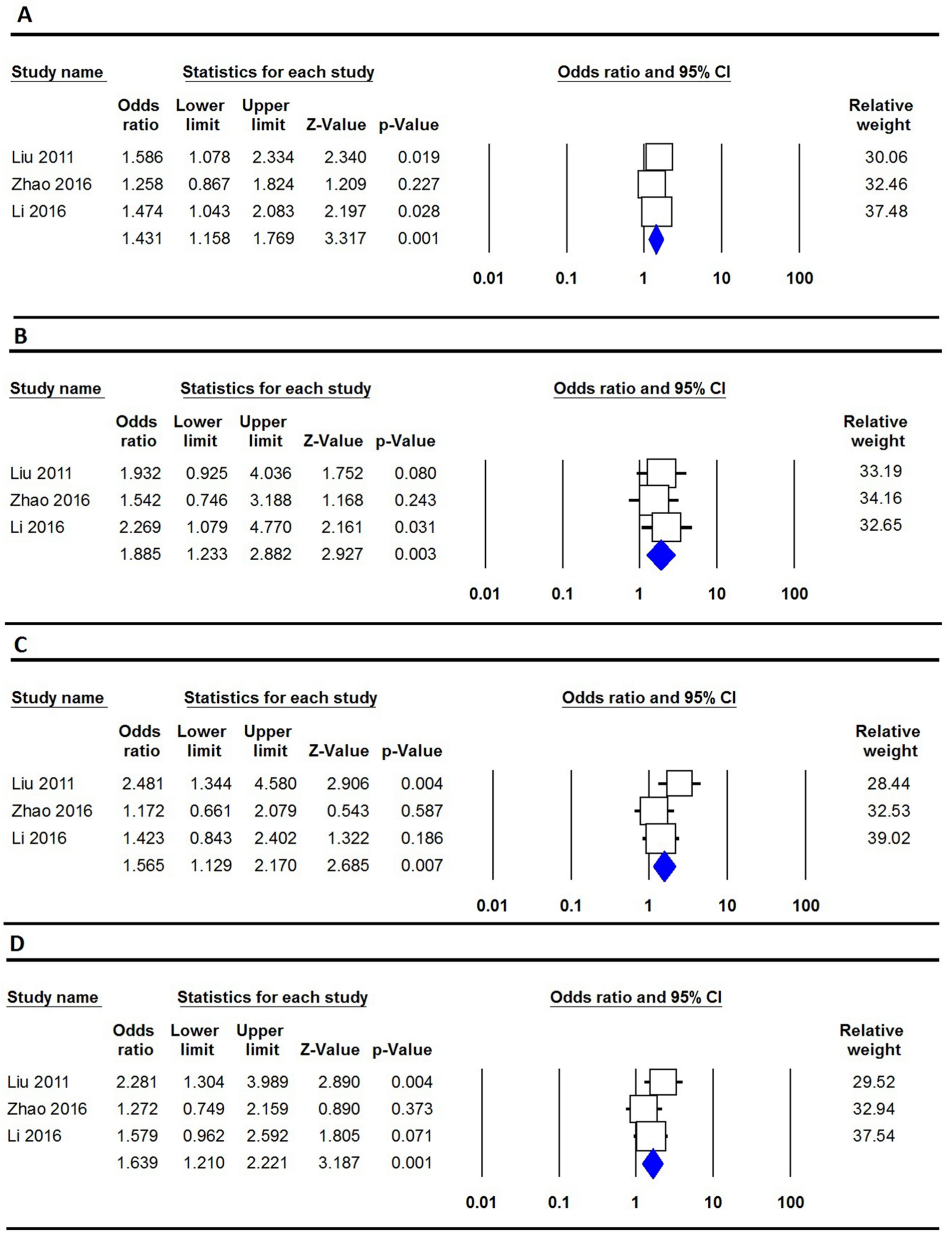
Forest plots for the association of COL9A1 rs1135056 polymorphism and risk of CTEV among Chinese. **A**: allele (G vs. A); **B**: homozygote (GG vs. AA); **C**: heterozygote (GA vs. AA); and **D**: dominant (GG + GA vs. AA)

The key findings regarding the association between COL9A1 polymorphisms and susceptibility to CTEV are concisely presented in Table 2. Overall, a notable connection was observed between the COL9A1 rs1135056 polymorphism and the susceptibility to CTEV under the homozygote model (GG vs. AA: OR = 1.487, 95% CI 1.023–2.160, *p* = 0.037, Fig. 2). Importantly, subgroup analysis based on the country of origin revealed a significant correlation among Chinese individuals across four genetic models: allele (G vs. A: OR = 1.431, 95% CI 1.158–1.769, *p* = 0.001, Fig. 3A), homozygote (GG vs. AA: OR = 1.885, 95% CI 1.233–2.882, *p* = 0.003, Fig. 3B), heterozygote (GA vs. AA: OR = 1.565, 95% CI 1.129–2.170, *p* = 0.007, Fig. 3C), and dominant (GG + GA vs. AA: OR = 1.639, 95% CI 1.210–2.221, *p* = 0.001, Fig. 3D). Regarding rs35470562, a significant correlation was observed across three genetic models: allele (A vs. G: OR = 0.667, 95% CI 1.038–2.131, *p* = 0.031), homozygote (AA vs. GG: OR = 2.600, 95% CI 1.156–5.850, *p* = 0.021), and recessive (AA vs. AG + GG: OR = 2.225, 95% CI 1.054–4.699, *p* = 0.036). However, the combined data analysis showed no significant correlation between COL9A1 rs592121 and the susceptibility to CTEV across all five genetic models in the overall population.

**Table 2:** Summary risk estimates for association between COL9A1 polymorphisms and risk of CTEV

Subgroup	Genetic Model	Type of Model	Heterogeneity		Odds Ratio (OR)				Publication Bias	
			I^2^(%)	*P*_H_	OR	95% CI	Z_OR_	*P*_OR_	*P*_Beggs_	*P*_Eggers_
**rs1135056**										
Overall	G vs. A	Random	78.66	0.003	1.170	0.782–1.751	0.763	0.446	0.734	0.516
	GG vs. AA	Fixed	48.62	0.120	1.487	1.023–2.160	2.082	0.037	1.000	0.171
	GA vs. AA	Random	84.48	≤ 0.001	1.141	0.557–2.338	0.360	0.719	0.734	0.640
	GG + GA vs. AA	Random	84.42	≤ 0.001	1.208	0.623–2.339	0.559	0.576	0.734	0.475
	GG vs. GA + AA	Fixed	0.00	0.582	1.334	0.946–1.880	1.645	0.100	0.734	0.389
Chinese	G vs. A	Fixed	0.00	0.683	1.431	1.158–1.769	3.317	0.001	1.000	0.934
	GG vs. AA	Fixed	0.00	0.764	1.885	1.233–2.882	2.927	0.003	0.296	0.033
	GA vs. AA	Fixed	38.95	0.194	1.565	1.129–2.170	2.685	0.007	1.000	0.560
	GG + GA vs. AA	Fixed	10.88	0.326	1.639	1.210–2.221	3.187	0.001	1.000	0.620
	GG vs. GA + AA	Fixed	0.00	0.660	1.467	0.997–2.157	1.946	0.052	1.000	0.283
**rs35470562**										
Overall	A vs. G	Fixed	0.00	0.667	1.487	1.038–2.131	2.162	0.031	NA	NA
	AA vs. GG	Fixed	0.00	1.000	2.600	1.156–5.850	2.310	0.021	NA	NA
	AG vs. GG	Fixed	0.00	0.832	1.299	0.777–2.175	0.997	0.319	NA	NA
	AA + AG vs. GG	Fixed	0.00	0.670	1.475	0.903–2.410	1.552	0.121	NA	NA
	AA vs. AG + GG	Fixed	0.00	1.000	2.225	1.054–4.699	2.098	0.036	NA	NA
**rs592121**										
Overall	C vs. T	Fixed	0.00	0.936	1.087	0.837–1.411	0.623	0.534	NA	NA
	CC vs. TT	Fixed	0.00	0.891	1.236	0.710–2.154	0.749	0.454	NA	NA
	CT vs. TT	Fixed	0.00	0.832	1.022	0.691–1.510	0.108	0.914	NA	NA
	CC + CT vs. TT	Fixed	0.00	0.851	1.067	0.737–1.543	0.344	0.731	NA	NA
	CC vs. CT + TT	Fixed	0.00	0.811	1.206	0.729–1.994	0.728	0.467	NA	NA

### Sensitivity analysis

A sensitivity analysis was conducted to assess whether the combined data were significantly affected by the presence of any individual study. This was achieved by systematically removing each study and recalculating the significance of the findings. The results showed that the fixed-effects and/or random-effects estimates remained largely consistent before and after the removal of each study, suggesting that the combined odds ratio estimates were stable. Additionally, a sensitivity analysis was performed by excluding studies in which the controls did not adhere to HWE. The analysis revealed that there was no heterogeneity observed before or after the exclusion of these studies.

### Heterogeneity test

As presented in Table 2, the current meta-analysis findings indicate a moderate to high level of variation among the included studies for the rs1135056 polymorphism under three genetic models overall. However, this variation was not observed for the rs35470562 and rs592121 polymorphisms. Consequently, we conducted a subgroup analysis based on the country of origin, revealing that the country of origin is a significant source of the variation observed for this specific polymorphism (Table 2). This study provides an interesting perspective and serves as a compelling example of how ethnicity can influence the variation observed between studies.

### Publication bias

The intuitive evaluation of publication bias was carried out by constructing a Begg’s funnel plot, while the quantitative analysis was performed using Egger’s test. This statistical method involved plotting the standard error of the natural logarithm of the odds ratios against the odds ratios for each study included in the meta-analysis. As per the commonly acknowledged interpretation, the identification of selection bias would be reflected in an asymmetric plot, indicating a significant influence on the overall meta-analysis. Nevertheless, the shapes of the Begg’s funnel plots concerning the rs1135056, rs35470562, and rs592121 polymorphisms, across all five genetic models, did not display any signs of publication bias. This lack of bias was further validated through the implementation of the Egger test, as illustrated in Table 2.

## Discussion

There exists a multitude of theories regarding the etiology and pathogenesis of CTEV. These theories primarily revolve around abnormal bone development, neuromuscular disease, soft tissue contracture, vascular abnormalities, intrauterine growth retardation, maternal smoking, conception month, birth season, and other factors. However, it should be noted that there is a dearth of substantial evidence supporting these theories [1, 46, 47]. It has been reported that the development of clubfoot involves various genes, such as PITX1, HOXD9, and TBX4, thereby emphasizing the intricate nature of genetic contributions to this condition [48, 49]. These findings serve to underscore that CTEV is influenced by multiple genetic variants, highlighting the significance of considering a comprehensive range of genetic factors when attempting to comprehend its genetic architecture. In several prior studies, the ICTEV susceptibility gene was mapped to the 6q12-13 region. Within this candidate region, several known genes are present, including COL9A1, PGM3, BAI3, COL19A1, and COL12A1. Notably, the COL9A1 gene is situated among these candidate genes within the aforementioned region [50, 51].

The Type IX collagen exhibits the capability to form associations with various molecules such as tissue inhibitors of matrix metalloproteinases, surface receptors, and growth factors present on the chondrocyte membrane. The significance of its function lies in its ability to stabilize the internal environment of articular cartilage and provide protection to it [52]. The α1 chain, which is a major component of type IX collagen, is found in hyaline cartilage [53]. The COL9A1 gene was originally identified by Kimura et al. in 1989 [54] and its location was determined to be at 6q12-q13 by Warman et al. in 1993 using fluorescence in situ hybridization [55]. Two transcripts of different lengths exist, with full lengths of 3704 bp and 2985 bp, and containing 38 and 32 exons, respectively. Current research suggests that mutations in the type IX collagen gene can lead to mild achondroplasia. Additionally, mutations in exon 9 of the COL9A1 gene have been linked to Kashin-Beck disease, congenital epiphyseal dysplasia, osteoarthritis, and Stickler syndrome. A study conducted by Shi et al. in 2015 involving 274 cases of Kashin-Beck disease and 248 disease-free children revealed a significant correlation between COL9A1 rs6910140 and the risk of this pathology [56]. However, other studies have reported inconsistent findings. For instance, Czarny-Ratajczak et al. in 2001 failed to establish a substantial correlation between genetic variants in the COL9A1 gene and the risk of multiple epiphyseal dysplasia in the Finnish population [57]. Similarly, Jakkula and colleagues (2005) also reported no statistically significant association between various cartilage collagen genes, such as COL2A1, COL9A1, COL9A2, and COL9A3, and the occurrence of osteoarthritis in infants [58].

In the present investigation, a collective sum of eight case-control studies, comprising a total of 833 cases of CTEV and 1280 control individuals, were incorporated. Four of the studies examined rs1135056, with a total of 432 cases of CTEV and 603 controls. Another two studies specifically investigated rs35470562, with 189 CTEV cases and 378 controls. Additionally, two studies focused on rs592121, comprising 212 CTEV cases and 299 controls. Our amalgamated data implies that the COL9A1 rs1135056 and rs35470562 polymorphisms exhibit a statistically significant association with susceptibility to CTEV. Nevertheless, no significant correlation was found between the rs592121 polymorphism and the risk of CTEV. Previously, various studies have reported that polymorphisms in the COL9A1 gene are associated with the risk of CTEV. In particular, the COL9A1 rs1135056 has been found to have a significant relationship with CTEV in both children and their biological mothers [54]. Furthermore, it has been observed that individuals with the GG genotype for rs592121 have a higher likelihood of developing CTEV compared to those with other genotypes [59]. Another study has also indicated that the AA genotype of COL9A1 rs35470562 is significantly linked to an increased risk of CTEV when compared to the GG genotype [60]. However, no significant association was established between the COL9A1 rs1135056 and rs592121 polymorphisms and CTEV in any of the genetic models tested [38]. These findings suggest that COL9A1 polymorphisms, particularly rs1135056 and rs35470562, may play a role in the development of CTEV.

In 2011, Liu et al. conducted an examination on the correlation between COL9A1 polymorphism and susceptibility to CTEV. In their analysis, which included 118 CTEV cases and 100 normal cases, the authors proclaimed that the COL9A1 protein is substantially overexpressed in patients with CTEV, and the rs1135056 polymorphism is associated with its pathogenesis [38]. In a distinct investigation carried out by Liu et al. in the year 2007, two variations of genetic makeup, specifically rs592121 and rs1135056, were evaluated within the COL9A1 gene in a total of 84 nuclear pedigrees related to individuals with ICTEV. The findings of the study revealed that there was an observed transmission disequilibrium in the pedigrees for the rs592121 and rs1135056 polymorphisms within the COL9A1 gene. Furthermore, the expression levels of COL9A1 mRNA were significantly elevated in patients with ICTEV compared to individuals without the condition [61]. In the year 2022, Raikwar et al. conducted a study on a dyad consisting of a mother and child to investigate the impact of two significant polymorphisms, namely rs592121 and rs1135056, at the COL9A1 gene on the development of CTEV. The results of their research revealed a potential link between the rs592121 gene polymorphism and an increased susceptibility to CTEV in patients [54]. In 2016, the study conducted by Zhao et al. investigated the possible association between three genetic polymorphisms, namely COL9A1 rs1135056, rs35470562, and rs592121, and the risk of CTEV in a sample of 87 cases and 174 controls recruited from the Fourth People’s Hospital of Shaanxi and the First Hospital of Yulin. The authors performed a conditional regression analysis and found that individuals with the AA genotype of COL9A1 rs35470562 exhibited a significantly higher risk of developing CTEV compared to those with the GG genotype (OR = 2.60, 95% CI = 1.06–6.32). Furthermore, when using a recessive model, it was observed that carriers of the rs35470562 AA genotype are at a greater risk for CTEV in comparison to individuals with either the GG or GA genotypes (OR = 2.23, 95%CI = 1.03–5.04). However, no significant relationship was found between the COL9A1 rs1135056 and rs592121 polymorphisms and CTEV in Chinese children [59]. In another investigation involving 692 cases and 1513 healthy controls within the Han Chinese population, Zhao et al. conducted an assessment on the impact of COL9A1 gene variations on the vulnerability to CTEV. The findings of the study demonstrated that while there was no correlation observed between the genotyped SNPs and CTEV, an indication of a gene-environment interaction emerged between the SNP rs6455357 and maternal alcohol consumption [62]. In 2016, Zhao et al. reported a lack of association between the COL9A1 rs1135056 and rs592121 polymorphisms and the risk of CTEV, yet a significant correlation was identified between the rs35470562 polymorphism and this particular disease [59]. Additionally, several investigations have genotyped the COL9A1 rs1135056, rs35470562, and rs592121 using RFLP-PCR and have proposed a positive correlation between COL9A1 polymorphisms and the risk of CTEV within the Chinese population. For instance, two studies published in 2016 have demonstrated that the COL9A1 rs1135056 polymorphism may have an influence on the development of CTEV in the Chinese population [63, 64].

To the best of our knowledge, this meta-analysis represents the initial aggregation of data on the role of COL9A1 polymorphisms in the development of CTEV. The analysis was conducted through a comprehensive search of the available literature. However, there are some limitations to this meta-analysis. Firstly, the analysis was constrained by a relatively small number of studies, which limited the statistical power and generalizability of the findings. Additionally, the quality of the included studies varied, with some having small sample sizes and potential biases. Furthermore, the analysis was limited to published studies, which raises the possibility of publication bias. Secondly, most of the studies focused on Chinese individuals, limiting our ability to evaluate the potential impact of COL9A1 polymorphisms on other ethnic groups. This weakness weakens the true association between COL9A1 polymorphisms and the risk of CTEV. Therefore, it is crucial to conduct large, multi-center studies with sufficient power to thoroughly investigate these associations in various ethnic groups. Thirdly, this meta-analysis only included published papers available in the online network, and it is possible that some unpublished studies with null results were overlooked, potentially biasing the pooled data. Fourthly, we were unable to obtain data on various confounding factors known to influence the development of CTEV, and a more rigorous analysis could be performed if the selected studies provided adjusted evaluations. Lastly, despite significant advancements, there are still numerous challenges in elucidating the genetic basis of CTEV. The complex interplay between genetic and environmental factors, as well as the phenotypic variability, pose a formidable challenge. The meta-analysis focused solely on the association between COL9A1 polymorphisms and CTEV, and did not investigate the potential influence of other genetic or environmental factors on the development of this condition due to limited original data from the included studies.

Our aggregated data indicates that the COL9A1 rs1135056 (c.1862 A > G, p.Gln621Arg) and rs35470562 (c.2050G > A, p.Glu441Lys) variations are significantly associated with susceptibility to CTEV. However, the rs592121 (c.1015T > C, p.Ser339Pro) polymorphism does not show the same association. Nevertheless, caution is warranted when interpreting the findings of this meta-analysis, as our results only allow for the examination of the effects of COL9A1 polymorphisms in Asian children with CTEV. Understanding the genetic basis of CTEV has important implications for clinical practice. Genetic testing can facilitate early diagnosis and risk assessment for families with a history of clubfoot. Identifying the responsible genes opens up the possibility of gene-targeted therapies and personalized treatment options, leading to improved patient outcomes. Therefore, further studies with larger sample sizes and sufficient statistical power across diverse ethnic groups are necessary to gain a better understanding of the role of COL9A1 polymorphisms in the development of CTEV and to consider the combined impact of genetic and environmental factors on its etiology.

## Data Availability

No datasets were generated or analysed during the current study.
